# 

**Published:** 1890-01

**Authors:** 


### CONTENTS.

ORIGINAL COMMUNICATIONS—

The Treatment of Contracted Bladder by Hot Water Dilatation. By I. S. Stone, M. D., Lincoln, Va. .... 651

The Treatment of Organic Urethral Stricture. By F. W. McRae, M. D., Atlanta, Ga. .... 659

Carcinoma of the Neck of the Uterus Complicating Pregnancy and Labor. By G. Heinricius .... 665

SOCIETY REPORTS—

Allegheny County Medical Society .... 670

Philadelphia Obstetrical Society .... 681

CORRESPONDENCE—

Our New York Letter .... 684

Letter from K. P. Moore, M. D., Macon, Ga. .... 689

REPORT OF CASES—

Left Inguinal Colotomy for Carcinoma of Rectum. Performed by J. McF. Gaston, M. D. .... 695

An Unusual Case .... 697

BOOK REVIEWS—

Diseases of the Nose and Throat. By Francke Huntington Bosworth, A. M., M. D. .... 698

A Text Book of Ophthalmology and Ophthalmoscopy. By Hermann Schmidt-Rimpler .... 699

Annual of the Universal Medical Sciences. Edited by Charles E. Sajons, M. D. .... 700

EDITORIAL—

Announcement .... i

The Atlanta Society of Medicine .... 702

A New Antiseptic .... 704

Items .... 705

